# Effects of preventive use of compression stockings for elderly with chronic venous insufficiency and swollen legs: a systematic review and meta-analysis

**DOI:** 10.1186/s12877-019-1087-1

**Published:** 2019-03-07

**Authors:** Kristin Thuve Dahm, Hilde Tinderholt Myrhaug, Hilde Strømme, Brynjar Fure, Kjetil Gundro Brurberg

**Affiliations:** 1 Division of Health Services, Norwegian Institute of Public Health, Box 4404 Nydalen, 0130 Oslo, Norway; 2 University of Oslo Medical Library, Rikshospitalet, Sognsvannsveien 20, 0372 Oslo, Norway; 3 Department of Internal Medicine, Central Hospital Karlstad, Rosenborgsgatan 9, 652 30 Karlstad, Sweden

**Keywords:** Elderly, Venous insufficiency, Compression stockings, Systematic review

## Abstract

**Background:**

Many home-dwelling elderly use medical compression stockings to prevent venous insufficiency, deep venous thrombosis, painful legs and leg ulcers. Assisting users with applying and removing compression stockings demands resources from the home based health services, but the effects are uncertain. This systematic review aims to summarize the effects of preventive use of medical compression stockings for patients with chronic venous insufficiency and swollen legs.

**Methods:**

We conducted a search in six databases (Epistemonikos, Cochrane Database of Systematic Reviews, MEDLINE, Embase, CENTRAL and CINAHL) in March 2018. Randomized controlled trials evaluating the preventive effects of European standard compression stockings class 3 or 2 for elderly with chronic venous insufficiency and swollen legs were included. Primary outcomes were thrombosis, leg ulcers and mobility. Secondary outcomes were other health related outcomes, e.g. pain, compliance.

We assessed risk of bias in the included studies and used the Grading of Recommendations Assessment, Development and Evaluation (GRADE) tool for evaluating the overall quality of evidence.

**Results:**

Five randomized controlled trials met the inclusion criteria. Comparing compression stockings class 2 to class 1, meta-analysis showed a reduction in leg ulcer recurrence at 12 months (RR 0.52; 95% CI 0.30 to 0.88). The quality of evidence was assessed as moderate by GRADE. One study (100 participants) did not detect a difference between compression stockings class 3 versus class 2 on ulcer recurrence after six months (RR 0.64; 95% CI 0.20 to 2.03). In another study, patients wearing class 3 compression stockings had lower recurrence risk compared with patients without stockings (RR 0.46; 95% CI 0.27 to 0.76) at six months and **(**RR 0.43; 95% CI 0.27 to 0.69) at 12 months. We found no difference between class 2 and class 1 stockings on subjective symptoms of chronic venous insufficiency or outcomes of vein thrombosis or mobility.

**Conclusion:**

Compression stockings class 2 probably reduce the risk of leg ulcer recurrence compared to compression stockings class 1. It is uncertain whether the use of stockings with higher compression grades is associated with a further risk reduction. More randomized controlled trials on vein thrombosis and mobility are needed.

**Electronic supplementary material:**

The online version of this article (10.1186/s12877-019-1087-1) contains supplementary material, which is available to authorized users.

## Background

Chronic venous insufficiency refers to a condition with impaired blood flow in the deep leg veins, and is usually caused by inadequate venous valves. The condition is characterized by symptoms like oedema, skin changes, fatigue, leg pain and a sensation of heaviness in the leg, and can be diagnosed by using ultrasound techniques to detect venous reflux and pooling of blood in deep leg veins [1]. Venous insufficiency may develop into chronic leg ulcer and deep vein thrombosis. Venous thrombosis may damage the valves, and symptoms and signs of chronic venous insufficiency following a deep vein thrombosis (DVT) are called post-thrombotic syndrome [2]. The Clinical-Etiology-Anatomy-Pathophysiology (CEAP) classification is an accepted standard for classifying chronic venous disorders. The chronic venous disorders are classified from C0 to C6 based on the severity of venous symptoms [1]. The prevalence of venous insufficiency varies considerably between genders, ethnic backgrounds and age groups [3]. A German cross-sectional study from 2008 included more than 3000 people aged 18 to 79 years, and estimated the overall prevalence of venous insufficiency to 31% [4]. The risk of venous insufficiency increases with age [5].

Physical activity, smoking cessation, weight reduction, leg elevation, anticoagulant drugs and medical compression stockings may reduce symptoms and prevent progress of chronic venous insufficiency [6]. External compression, such as medical compression stockings, may reduce oedema and swelling, and improve microcirculation [7]. Medical compression stockings reach the knee or the hip and usually exert a pressure of 20 to 40 mmHg. Different standards exist, but according to the European standard, compression stockings are categorised into four classes [8]. Class 1 stockings exert pressures below 20 mmHg and are used to prevent oedema. Class 2 stockings exert pressures between 20 and 30 mmHg and are used in the prevention of venous insufficiency and varicose veins. Class 3 stockings result in high compressions between 30 and 40 mmHg and are used for chronic venous insufficiency, whereas class 4 stockings exert very high compression above 40 mmHg and are primarily used in the treatment of lymphoedema.

Two studies [9, 10] support the use of compression stockings in the treatment of chronic venous disease in patients under 70 years old, whereas the effectiveness is not known in older populations. Systematic reviews about post-thrombotic syndrome [11–14] and pain [11] in patients with deep venous thrombosis show uncertain effects. Systematic reviews published in 2013 and 2014 [15, 16] question the effects of preventive use of compression stockings for patients with venous insufficiency, but a preliminary search showed that these systematic reviews were no longer up to date. Elderly patients with chronic venous insufficiency and multimorbidity are of particular interest because they frequently need assistance from home care personnel to administer compression stockings. In this work, we undertake an updated systematic review to investigate the preventive effects of medical compression stockings for elderly patients with chronic venous insufficiency and swollen legs.

## Methods

This systematic review follows the recommendations of the Cochrane handbook of systematic reviews of interventions [17]. The protocol of this systematic review was registered in the international prospective register of systematic reviews (PROSPERO) with registration number CRD42018092944.

### Search methodology

An information specialist (HS) planned and performed a systematic search in the following databases: Epistemonikos, Cochrane Database of Systematic Reviews, MEDLINE, Embase, CENTRAL and CINAHL in March 2018. We used a combination of subject headings and text words for venous insufficiency and compression stockings. In addition, searches were made in WHO International Clinical Trials Registry and ClinicalTrials.gov for ongoing studies in August 2018. The search strategies were adapted to each database as presented in Additional file 1.

### Study selection, data extraction and analysis

We included systematic reviews (SRs) and randomized controlled trials (RCTs) according to the following criteria: (a) study population of elderly (≥70 years) with venous insufficiency and swollen legs without recent (≤ 2 years) deep vein thrombosis; (b) evaluating the preventive effects of European standard compression stockings class 2 or 3; (c) compared to a different class of compression stockings, other interventions to promote venous backflow or no intervention; (d) assessed on thrombosis, leg ulcer and mobility (primary outcomes) or other health related outcomes such as pain, discomfort, quality of life or post-thrombotic syndrome (secondary outcomes). Compliance was not defined as an outcome in the original protocol, but following feedback, we included compliance as a secondary outcome post hoc.

Two reviewers independently assessed the titles and abstracts of records identified by the search. Records appearing to meet the inclusion criteria and those with insufficient details were obtained in full text. Two reviewers independently assessed the full text publications according to a pre-defined inclusion form. Any discrepancies were resolved by consensus.

The first author (KTD) described the included trials with regard to population, intervention, comparison, outcome and main results in tables. Another reviewer (HTM) checked the extracted information. Two reviewers (KTD, HTM) independently assessed the methodological quality of included studies using the Risk of Bias assessment tool [17].

We conducted meta-analysis in Review Manager (RevMan5.3) software [18], when studies were sufficiently similar in terms of design, population, interventions and outcomes. We calculated relative risk (RR) for dichotomous outcomes and mean difference (MD) for continuous outcomes, both with 95% confidence interval (CI). We used a random effects model to account for pooling effects due to the clinical heterogeneity of the included studies. Double-data entries were performed. We planned to do subgroup analysis based on population and degree of compression, but due to the small number of studies, this was not feasible.

We assessed the quality of the evidence by using the Grading of Recommendations Assessment, Development and Evaluation (GRADE) [19]. The assessment involves within-study risk of bias, directness of evidence, inconsistency of effect estimates (heterogeneity), precision of effect estimate and risk of publication bias. The GRADE assessment indicates the extent to which we can have confidence in the effect estimate. Confidence of the effect estimates were described as high, moderate, low and very low (Table 1).

**Table 1:** Confidence in effect estimates with interpretation

Quality of evidence	Interpretation
High	Further research is very unlikely to change our confidence in the estimate of effect.
Moderate	Further research is likely to have an important impact on confidence in the estimate of effect and may change the estimate.
Low	Further research is very likely to have an important impact on confidence in the estimate of effect and is likely to change the estimate.
Very low	Any estimate of effect is very uncertain.

## Results

**Fig. 1:**
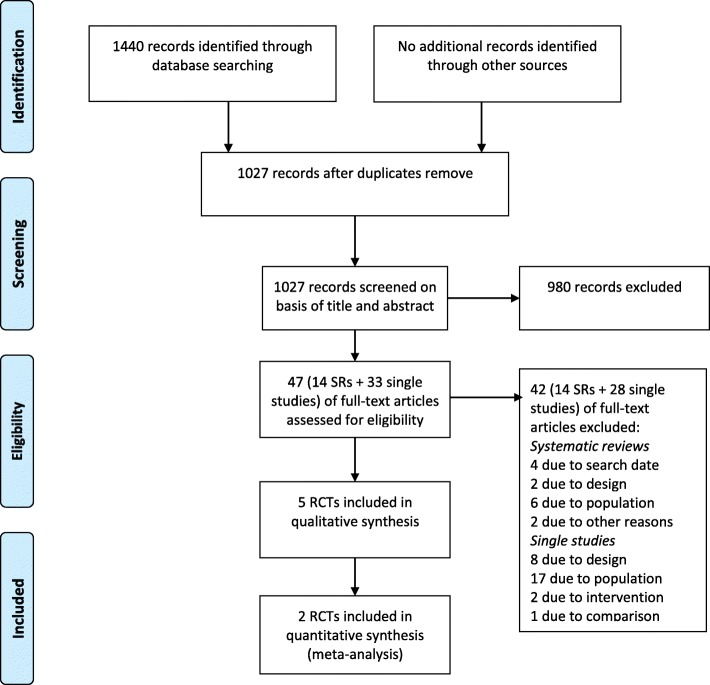
PRISMA Flowchart of the study selection process

The result of the study selection is shown in Fig. 1. The search yielded 1027 unique references, and 47 references were retrieved and reviewed in full text. Five randomized controlled trials were included. We found no relevant ongoing studies. The studies were published between 1997 and 2013 and included a total of 684 participants with chronic venous insufficiency. Two studies were from Australia [20, 21], one from England [22], one from Ireland [23] and one from Sweden [24].

### Description of studies

An overview of included studies is presented in Table 2. Four studies [20–23] with 653 participants examined the effects of compression stockings treatment on ulcer recurrence for patients with recently healed venous leg ulcers. Four studies [20, 21, 23, 24] used European standard for defining compression classes, and they compared different grades of compression. One study [22] used British standard and we redefined this to European standard. Two of the included studies [22, 23] reported education related to skin care and the use of compression stockings as part of the intervention, and two studies [21, 23] included self-reported assessment of compliance as part of the provided intervention.

**Table 2:** Overview of included studies

Study Setting, Country	Population	Intervention*	Comparison	Results Primary outcomes	Results Secondary outcomes
Patients with healed venous leg ulcers					
*Kapp 2013 (20)* Home nursing Australia	*n* = 100 Age: 79 ± 10 Recently healed venous leg ulcer	Class 3 stockings (34–46 mmHg) below knee length	Class 2 stockings (23–32 mmHg) below knee length	*Ulcer recurrence:* 6 months RR: 0.64 (0.20 to 2.03)	*Compliance 6 months* Class 3: 17/44 Class 2: 35/49 RR: 0.54 (0.36 to 0.82)**
*Clarke-Moloney 2012 (23)* Hospital and community leg ulcer clinic Ireland	*n* = 100 Age: 69 ± 11 Recently healed venous leg ulcer	Class 2 stockings (23–32 mmHg) knee or thigh length	Class 1 stockings (18–21 mmHg) knee or thigh length	*Ulcer recurrence:* 12 months RR: 0.59 (0.23 to 1.49)	*Compliance 12 months* Class 2: 45/50 Class 1: 43/49 RR: 1.03 (0.89 to 1.18)
*Nelson 2006 (22)* Hospital leg ulcer clinic England	*n* = 300 Age:64 ± 12 Recently healed venous leg ulcer	Class 2 stockings (25–35 mmHg) knee or thigh length	Class 1 stockings (18–24 mmHg) knee or thigh length	*Ulcer recurrence:* 12 months RR: 0.47 (0.25 to 0.91)** Five years RR: 0.82 (0.61 to 1.12)	*Compliance 5 years* Class 2: 86/149 Class 1: 108/151 RR: 0.81 (0.68 to 0.96)**
*Vandongen 2000 (21)* Hospital leg ulcer clinic Australia	*n* = 153 Age: 67 [37–85] Venous leg ulcer healed for 2 weeks. Patients had to apply the stockings themselves	Class 3 stockings (35–45 mmHg) below knee length	No compression stockings	*Ulcer recurrence:* 6 months RR: 0.46 (0.27 to 0.76)** 12 months RR: 0.43 (0.27 to 0.69)**	*Compliance not reported*
Patients with venous insufficiency					
*Jungbeck 1997 (24)* Vascular surgery dept. Sweden	*n* = 31 Age: [27–82] Chronic venous insufficiency grade II	Class 2 stockings (23–30 mmHg) below knee length	Class 1 stockings (13–20 mmHg) below knee length	Not reported	*Compliance not reported* *Subjective symptoms 8 weeks* ^*1*^ Class 2 (*Median % on VAS)*: Before: 46.1%, after: 14.8% Class 1 (*Median % on VAS)*: Before: 43.8%, after: 15.6%

*We used the European standard of compression stockings

**Indicates statistically significant result

^1^Pain, ankle swelling, tired legs, restless legs and night cramps

One study included patients with signs of chronic venous insufficiency [20], whereas another study included patients with lipodermatosclerosis [21]. In two studies [20, 22] about 70% of the participants were women. Comorbidity and details about the application of the compression stockings were poorly reported in the included studies. However, in one study [21] the participants had to apply the compression stockings themselves. The fifth study [24] included 31 participants with chronic venous insufficiency grade II, comparing compression stockings class 2 and compression stockings class 1.

### Risk of bias

**Fig. 2:**
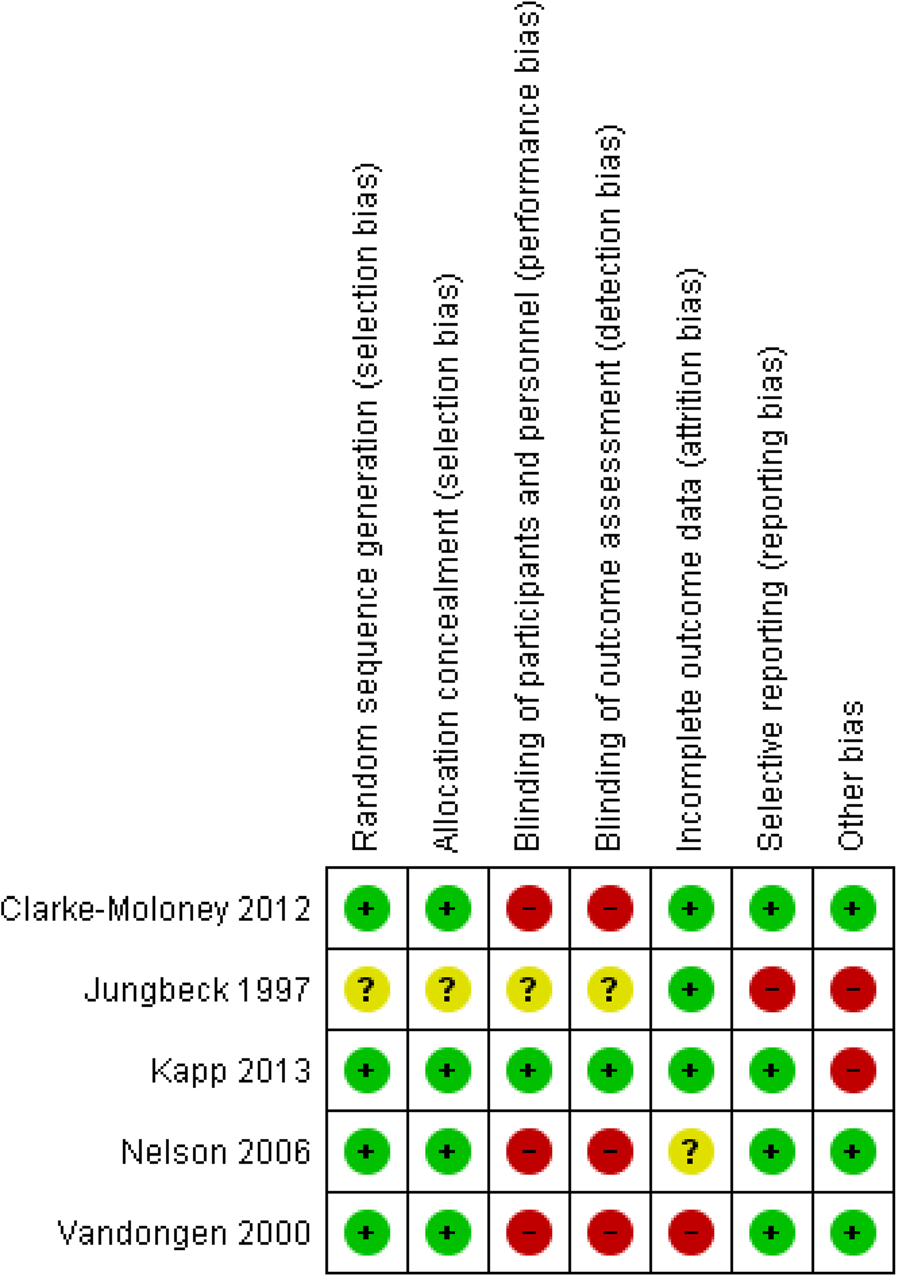
Risk of bias

The risk of bias assessments are summarized in Fig. 2. Four studies had low risk of selection bias. Three studies had high risk of performance and detection bias, whereas three studies had low risk of attrition bias, four studies had low risk of reporting bias and two studies had other biases (Fig. 2).

### Effects on leg ulcer recurrence

**Fig. 3:**
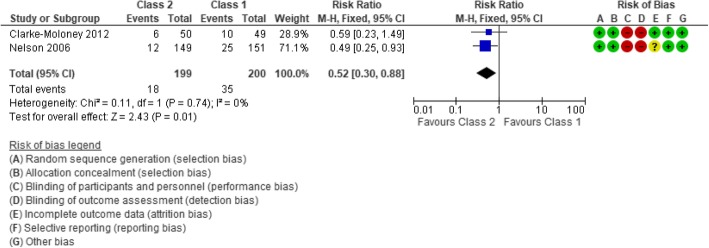
Forest plot ulcer recurrence compression stockings class 2 vs class 1

Four trials [20–23] examined the effects of compression stockings treatment on ulcer recurrence for patients with healed venous leg ulcers. We were able to conduct a meta-analysis (Fig. 3) of two trials comparing two different strengths of compression stockings on the risk of ulcer recurrence [22, 23]. Ulcer recurrence was defined as epithelial breakdown anywhere below the knee lasting more than four weeks and requiring bandage treatment in one study [23], and as a skin break failed to heal in six weeks in the other study [22]. The meta-analysis included data from 399 participants and showed that compression stockings class 2 significantly reduced leg ulcer recurrence compared to compression stockings class 1 (RR 0.52; 95% CI 0.30 to 0.88) at 12 months. Hence, if 175 per 1000 patients receiving class 1 compression stockings experience ulcer recurrence, the corresponding number among patients who receive class 2 stockings will be 91 per 1000. We have moderate confidence in the effect estimate (Table 3).

**Table 3:** Quality of evidence of ulcer recurrence

Outcome	Illustrative comparative risk		Relative effect (95% CI)	No of participants (studies)	Quality of evidence (GRADE)
	Assumed risk Control	Corresponding risk Compression stockings			
Comparison 1: Class 2 compared to class 1 (European standard)					
Ulcer recurrence 12 months	175 per 1000	84 fewer per 1000 (from 21 fewer to 122 fewer)	RR 0.52 (0.30, 0.88)	399 [2]	Moderate^1^ ⊕ ⊕ ⊕⊙
Comparison 2: Class 3 compared to class 2 (European standard)					
Ulcer recurrence 6 months	143 per 1000	51 fewer per 1000 (from 114 fewer to 147 more)	RR 0.64 (0.20, 2.03)	93 [1]	Very Low^2^ ⊕ ⊙ ⊙ ⊙
Comparison 3: Class 3 (European standard) compared to no stockings					
Ulcer recurrence 12 months	543 pr. 1000	310 fewer per 1000 (from 168 fewer to 397 fewer)	RR 0.43 (0.27, 0.69)	153 [1]	Very Low^3^ ⊕ ⊙ ⊙ ⊙

^1^Downgraded one level due to high risk of bias, ^2^Downgraded three levels due to severe impression (very wide confidence intervals, low compliance and few participants) and lack of consistency since it is only one trial, ^3^Downgraded three levels due to high risk of bias, impression (few participants) and lack of consistency since it is only one trial

Kapp et al. [20] compared the effects of compression stockings class 3 versus class 2. Healing date and anatomical location were used to determine time to ulcer recurrence and number of wounds, but the authors were not able to detect a difference in ulcer recurrence between the groups after six months (RR 0.64; 95% CI 0.20 to 2.03; 100 participants). Vandongen et al. [21] compared compression stockings class 3 to no compression stockings, and found that patients who wore stockings were at lower risk of ulcer recurrence after six months (RR 0.46; 95% CI 0.27 to 0.76; 153 participants) and 12 months **(**RR 0.43; 95% CI 0.27 to 0.69). We have very low confidence in these effect estimates (Table 3).

### Effects on vein thrombosis and mobility

None of the included studies reported risk of vein thrombosis or mobility.

### Effects on secondary outcomes

Jungbeck et al. [24] compared compression stockings class 2 and class 1 in 31 patients with chronic venous insufficiency grade II. The effects were assessed measuring foot volume and subjective symptoms (e.g. pain, ankle swelling, tired legs, restless legs and night cramps) after eight weeks. Subjective symptoms were assessed by visual analogue scale (VAS). The authors were not able to detect a difference between class 2 and class 1 stockings on subjective symptoms after eight weeks, nor did they find a difference in foot volume or reflux values between the two groups. As the latter results were based on a single study with few participants and high risk of bias, we have very low confidence in these effect estimates.

## Discussion

In this systematic review, we aimed to summarize the preventive effects of medical compression stockings for patients with chronic venous insufficiency and swollen legs. We included five randomized controlled trial. The main finding is that compression stockings class 2 probably reduce the risk of leg ulcer recurrence compared to compression stockings class 1. One included study [20] suggests that stockings of higher compression (class 3) were better than medium compression (class 2), but the study only included 100 participants and overall evidence was assessed as having low quality. Therefore, it remains uncertain whether the use of stockings with higher compression grades is associated with a further risk reduction. Moreover, it is uncertain whether the use of compression stockings reduces subjective symptoms and foot volume for patients with chronic venous insufficiency. We found no studies investigating the preventive use of compression stockings for patients with venous insufficiency or swollen legs on vein thrombosis or mobility.

The results we present on risk of ulcer recurrence are in accordance with the review of Nelson et al. [15]. The evidence is sparse, but class 2 compression stockings seem to be more effective than lower class stockings in the prevention of ulcer recurrence. Consistent with our findings, another review [16] states that the evidence is too sparse to allow firm conclusions about the effects of compression stockings for the initial treatment of varicose veins in patients without ulceration.

Two additional primary studies [9, 10] have shown that compression stockings are effective in reducing pain and symptoms in patients younger than 70 years suffering from chronic venous insufficiency. Moreover, a randomized controlled trial investigating the effects of progressive compression stockings (compression with maximal pressure at calf) [25] reported that progressive compression stockings (10 mmHg at ankle, 23 mmHg at upper calf) were more effective than ordinary compressive stockings (30 mmHg at ankle, 21 mmHg at upper calf) in reducing pain and heavy legs. However, the patients in the latter studies were too young to be included in our review, and the applicability to a geriatric population can be questioned.

The available evidence suggests that compression stockings may play a role in the prevention of ulcer recurrence, but the evidence has limitations. In addition to lack of blinding, attrition bias associated with incomplete outcome assessment and poor patient compliance reduces the quality of evidence. Patient compliance with the recommended regimen varies between studies and between treatment groups and it seems like the compliance rates decrease for stockings with higher compression grades. Three of the included studies did not report reasons for noncompliance [20–22], whereas one study reported that noncompliance was explained by tightness, inability to apply or remove the compression stockings and skin sensitivity [23]. The same study reported that poor compliance was associated with lower effect [23], but these findings were contradicted by studies reporting that the overall results did not change significantly when non-compliant patients were excluded from the analysis [20, 22].

It is reasonable to expect that poor compliance not only impact the effect of compression stockings, but also poses a challenge in ordinary practice. Healthcare professionals should focus on methods that improve patient compliance. Compression stockings may be a resource-demanding intervention, as elderly people with chronic venous insufficiency often need assistance from home care personnel to administer the stockings. Health professionals in close dialogue with each individual patient should evaluate the need for compression stockings before and during the treatment, because of the personnel cost, and the sparse and inconsistent body of the evidence.

A major strength of this systematic review is the extensiveness of the systematic search. Even though the search was comprehensive, only five randomized trials were included and no relevant ongoing studies were found. It is a limitation that the quality of the evidence was graded from moderate to very low, implying there is a need for further research on these topics before we can make a firm conclusion about the effects of preventive use of compression stockings.

Based on this systematic review, the prevalence of venous disease and the resources associated with the treatment [3], the research activity should continue to target this very important issue, preferably with more well-designed RCTs. In particular, there is a need for further studies about the preventive use of compression stockings for elderly patients with venous insufficiency and swollen legs. It is important to measure outcomes such as vein thrombosis and mobility in addition to leg ulcers.

## Conclusions

Based on the results of this systematic review, medical compression stockings probably reduce leg ulcer recurrence up to one year in elderly people, but the effect after one year is unclear. However, the evidence of initial treatment with compression stockings in patients with venous insufficiency or swollen legs is lacking.

## Additional file

Additional file 1: Search strategies. (PDF 263 kb)

## Abbreviations

CI: Confidence interval
GRADE: Grading of Recommendations Assessment Development and Evaluation
RCT: Randomized controlled trial
RR: Risk ratio
SR: Systematic review
VAS: Visual analogue scale
