# Supplementary material for: Effects of preventive use of compression stockings for elderly with chronic venous insufficiency and swollen legs: a systematic review and meta-analysis
Source: BMC Geriatr. 2019 Mar 7;19:76. doi: 10.1186/s12877-019-1087-1 (PMC6407277; doi:10.1186/s12877-019-1087-1)
Supplement: Supplementary file 1 — Search strategies. (PDF 263 kb) [file 12877_2019_1087_MOESM1_ESM.pdf]

Additional file 1. Search strategies

Database: **Embase** <1974 to 2018 February 23>, **Ovid MEDLINE(R)** Epub Ahead of Print, In-Process & Other Non-Indexed Citations, Ovid MEDLINE(R) Daily and Ovid MEDLINE(R) <1946 to Present> Search date: 26.02.2018

1 exp Venous Insufficiency/ or exp Varicose Veins/ or (edema/ and exp Lower Extremity/): hits 86671

2 (((venous or vein*) adj3 (insuff* or incompeten* or damage* or disease*)) or ((varicose or venous or stasis or leg or crural or cruris or (low* adj (extremit* or limb*))) adj1 vein*) or ((edema* or oedema*) adj3 (venous or leg or legs or foot or feet or ankle* or (low* adj (extremit* or limb*))))).tw,kw,kf.: hits: 57421

3 1 or 2 117286

4 Stockings, Compression/ or Compression Bandages/: hits: 5695

5 ((stocking* or sock* or hosiery or legging* or bandag* or wrap* or dressing* or garment*) adj3 (compress* or elastic or support* or anti-embolic or antiembolic or antithrombosis or thromboembolic or anti-thrombosis or thromboprophyla*)).tw,kw,kf.: hits 11027

6 4 or 5 14217

7 Meta Analysis.pt. or "Meta-Analysis as Topic"/ or (Review.pt. and (pubmed or medline).ti,ab.) or ((systematic* or literature) adj3 (overview or review* or search*)).ti,ab,kf. or (meta-anal* or metaanal* or meta-regression* or umbrella review* or overview of reviews or review of reviews or (evidence* adj2 synth*) or synthesis review*).ti,ab,kf.: hits 1088863

8 ((randomized controlled trial or controlled clinical trial).pt. or (randomi?ed or placebo or randomly).ab. or trial.ti. or clinical trials as topic.sh.) not (Animals/ not Humans/): hits 2239041

9 7 or 8: hits 3139235

10 3 and 6 and 9 use ppez: hits 487

11 exp vein insufficiency/ or ankle edema/ or foot edema/ or leg edema/: hits 23083

12 (((venous or vein*) adj3 (insuff* or incompeten* or damage* or disease*)) or ((varicose or venous or stasis or leg or crural or cruris or (lower adj (extremit* or limb*))) adj1 vein*) or ((edema* or oedema*) adj3 (venous or leg or legs or foot or feet or ankle* or (low* adj (extremit* or limb*))))).tw,kw.: hits 55546

13 11 or 12: hits 69613

14 compression stocking/ or compression bandage/ : hits 5998

15 ((stocking* or sock* or hosiery or legging* or bandag* or wrap* or dressing* or garment*) adj3 (compress* or elastic or support* or anti-embolic or antiembolic: hits 10998

or antithrombosis or thromboembolic or anti-thrombosis or thromboprophylaxis)).tw,kw.

16 14 or 15: hits 14367

17 "Meta Analysis"/ or "Systematic Review"/ or (review and (pubmed or medline)).ti,ab,kw. or ((systematic* or literature) adj3 (overview or review* or search*)).ti,ab. or (meta-anal* or metaanal* or meta-regression* or umbrella review* or overview of reviews or review of reviews or (evidence* adj2 synth*) or synthesis review*).ti,ab.: hits 1108607

18 (exp Clinical trial/ or Randomized controlled trial/ or Randomization/ or Single blind procedure/ or Double blind procedure/ or Crossover procedure/ or Placebo/ or Prospective study/ or (Randomi?ed controlled trial* or RCT or ((allocated or allocation) adj2 random*) or ((single or double or treble or triple) adj blind*) or placebo*).tw.) not (Case study/ or Abstract report/ or Case report.tw. or (Animal/ not Human/)): hits 3204996

19 17 or 18: hits 4107355

20 13 and 16 and 19 use oemezd: hits 560

21 10 or 20: hits 1047

22 remove duplicates from 21

CINAHL Search date: 26.02.18

Query Limiters Results

S1 (MH "Venous Insufficiency+" OR MH "Varicose Veins+" OR (MH "Edema" AND MH "Lower Extremity+")): hits 3,369

S2 (((venous OR vein) N2 (insuff* OR incompeten* OR damage* OR disease*)) OR ((varicose OR venous OR stasis OR leg OR crural OR cruris OR (lower W0 extremit*)) W0 vein*) OR ((edema* OR oedema*) N2 (venous OR leg OR legs OR foot OR feet OR ankle* OR (low* W0 (extremit* OR limb*))))): hits 2,524

S3 (MH "Compression Garments" OR MH "Elastic Bandages"): hits 1,689

S4 ((stocking* OR sock* OR hosiery OR legging* OR bandag* OR wrap* OR dressing* OR garment*) N2 (compress* OR elastic OR support* OR anti-embolic OR antiembolic OR antithrombosis OR thromboembolic OR anti-thrombosis OR thromboprophylaxis)): hits 2,439

S5 (((MH "Random Assignment") or (MH "Random Sample+") or (MH "Crossover Design") or (MH "Clinical Trials+") or (MH "Comparative Studies") or (MH "Control (Research)+") or (MH "Control Group") or (MH "Factorial Design") or (MH "Quasi-Experimental Studies+") or (MH "Placebos") or (MH "Meta Analysis") or (MH "Sample Size") or (MH "Research, Nursing") or (MH "Research Question") or (MH "Research Methodology+") or (MH "Evaluation Research+") or (MH "Concurrent Prospective Studies") or (MH "Prospective Studies") or (MH "Nursing Practice, Research-Based") or (MH "Solomon Four-Group

Design") or (MH "One-Shot Case Study") or (MH "Pretest-Posttest Design+") or (MH "Static Group Comparison") or (MH "Study Design") or (MH "Clinical Research+")) or (clinical nursing research or random* or cross?over or placebo* or control* or factorial or sham* or meta?analy* or systematic review* or blind* or mask* or trial*) OR ((((systematic* OR integrative OR comprehensive*) N3 (review* OR bibliographic* OR literature))) OR ((information OR data) N2 (synthesis OR extract*)) OR (meta-analy* or metaanaly*) OR (medline or pubmed or psyclit or cinahl or psycinfo or “web of science” or scopus or embase) OR (MH “Systematic Review”) or (MH “Meta Analysis”))): hits 1,539,591

S6 ((S1 OR S2) AND (S3 OR S4) AND S5): hits 367

S7 S6 Limiters - Exclude MEDLINE records: hits 148

((information OR data) N2 (synthesis OR extract*)) OR (meta-analy* or metaanaly*) OR (medline or pubmed or psyclit or cinahl or psycinfo or “web of science” or scopus or embase) OR (MH “Systematic Review”) or (MH “Meta Analysis”))): hits 1,539,591

S6 ((S1 OR S2) AND (S3 OR S4) AND S5): hits 367

S7 S6 Limiters - Exclude MEDLINE records: hits 148

Cochrane Library (Cochrane Reviews, Trials) Search date: 26.02.2018

#1 ([mh "Venous Insufficiency"] or [mh "Varicose Veins"] or ([mh ^edema] and [mh "Lower Extremity"])): hits 1442

#2 (((venous or vein*) near/3 (insuff* or incompeten* or damage* or disease*)) or ((varicose or venous or stasis or leg or crural or cruris or (low* next (extremit* or limb*))) near/1 vein*) or ((edema* or oedema*) near/3 (venous or leg or legs or foot or feet or ankle* or (low* next (extremit* or limb*))))):ti,ab,kw: hits 3290

#3 ([mh ^"Stockings, Compression"] or [mh ^"Compression Bandages"]): hits 368

#4 ((stocking* or sock* or hosiery or legging* or bandag* or wrap* or dressing* or garment*) near/3 (compress* or elastic or support* or anti-embolic or antiembolic or antithrombosis or thromboembolic or anti-thrombosis or thromboprophyla*)):ti,ab,kw 1507

#5 (#1 or #2) and (#3 or #4) in Cochrane Reviews (Reviews and Protocols) and Trials hits: 450

Epistemonikos: Search date: 26.02.2018

Search Title OR Abstract: (("venous insufficiency" OR "venous insufficiencies" OR "vein insufficiency" OR "vein insufficiencies" OR "insufficient vein" OR "insufficient veins" OR "venous incompetence" OR "venous disease" OR "vein disease" OR "vein incompetence" OR "vein damage" OR "venous damage" OR "damaged vein" OR "damaged veins" OR "vein

varicosis" OR "varicose vein" OR "varicose veins" OR ((edema OR oedema) AND (leg* OR "lower extremity" OR "lower extremities" OR "lower limb" OR "lower limbs"))) AND (stocking* OR sock* OR hosiery OR legging* OR bandag* OR wrap* OR dressing* OR garment*))

Broad Synthesis: hits 1

Structured Summary: hits 3

Systematic Review: hits 31

ClinicalTrials.gov

Date searched: 30 August, 2018

Number of hits: 15

Condition or disease: Venous insufficiency of leg

Other terms: stocking OR stockings OR sock OR socks OR hosiery OR legging OR leggings OR bandage OR bandages OR wrapping OR wraps OR wrap OR dressing OR dressings OR garment OR garments

Study type: Interventional Studies (Clinical Trials)

Age group: Older Adult (65+)

WHO International Clinical Trials Registry Platform

Date searched: 30 August, 2018

In the Condition: venous insufficienc* OR vein insufficienc* OR insufficient vein* OR venous incompetence OR venous disease OR vein disease OR vein incompetence OR vein damage OR venous damage OR damaged vein* OR vein varicosis OR varicose vein*

In the Intervention: stocking* OR sock* OR hosiery OR legging* OR bandag* OR wrap* OR dressing* OR garment*

Number of hits: 17
